# Characteristics and spending patterns of high-cost child patients: findings from Fujian in China

**DOI:** 10.1186/s12889-024-18246-x

**Published:** 2024-05-15

**Authors:** Xiaobo Peng, Ningning Guo

**Affiliations:** 1 School of Economics, Central University of Finance and Economics, Shahe Higher Education Park, Changping District, Beijing, China; 2 Kelley School of Business, Indiana University, Bloomington, USA

**Keywords:** Children, Health expenditures, Persistence and concentration, Risk protection, China

## Abstract

**Background:**

The health condition during childhood has been shown to influence an individual’s health and socioeconomic status in adulthood. Understanding the concentration and persistence patterns in children’s healthcare expenditures is crucial for providing risk protection and promoting the well-being of children. Studies regarding the concentration and persistence of health expenditures have focused mainly on elderly individuals in developed regions. To gain insights into factors that contribute to childhood health expenditures, this article examined children with high costs (that is, in the top 10% of the expenditure distribution) and explored the characteristics and spending patterns that distinguished them from other patients in the context of the largest developing economy—China.

**Methods:**

By using a unique individual-level administrative claims dataset over a 5-year observation period, this study identified spending concentrations and the proportion of children whose costs remained high over five years using a linear probability model and logit regression analysis.

**Results:**

Teenagers from 12 to 17 years old were more likely to persist in the high-cost group than any other age groups in the study. Pediatric complex chronic conditions and other severe health ailments were predictive factors for entry into and persistence in the high-cost category. More than half of the total health expenditures were attributed to children in the top 10% expenditure group. In addition, risk protection and healthcare insurance support for high-cost children was found to be inadequate, particularly for children from low-income families.

**Conclusions:**

Healthcare support for children impacts individual development and family financial status. This study described the characteristics and spending patterns of children patients in the largest developing country. The fact that over half of total expenditures are concentrated toward 10% of patients makes it valuable to consider relevant support for this group, especially for families whose medical costs are higher than income.

**Supplementary Information:**

The online version contains supplementary material available at 10.1186/s12889-024-18246-x.

## Background

The health conditions during childhood have been shown to influence long-term socioeconomic outcomes and adult health status for an individual. Investing in health during the early stages of an individual’s life is thus regarded as highly valuable [1]. An effective strategy for investing in health involves obtaining health insurance. Several countries have established government-funded health insurance programs, such as the Children’s Health Insurance Program in the U.S. Additionally, countries with universal health insurance typically offer substantial subsidies for childhood healthcare. This practice is observed in the Netherlands, Germany, Sweden, Taiwan (China), Japan and South Korea [2–4].

Compared to developed countries, developing countries have not paid enough attention to medical support for children [5]. In China, despite achieving universal health insurance^1^, the current health insurance system does not have specific coverage targeted for children. Children have distinctive medical service needs and disease types compared to adults [3]. In particular, a small percentage of children can consume a large portion of health resources, a situation that can last for years [6]. The occurrence of illness in children may impose financial stress and, in turn, could lead to poverty for their families [7].. Therefore, it is important to understand the patterns of concentration and persistence in children’s healthcare expenditures in order to provide risk protection and promote children’s health. This article examined children with high costs (that is, in the top 10% of the expenditure distribution) and explored the characteristics and spending patterns that distinguished them from other patients in the context of the largest developing economy—China.

A robust set of research conducted in developed countries and regions suggest that a small percentage of the population accounts for a large share of total health care expenditures [8] and that these patients persistently exhibit high spending over time [e.g., 9, 10]. Studies regarding the concentration and persistence of healthcare spending mainly focused on elderly individuals or on other adults. Eichner (1998) found the persistence of healthcare expenditures for employees in the U.S., and this phenomenon would intensify as age rose [10]. Later, Monheit (2003) confirms that 30% and 45% of the top 5% and 10% of medical spending groups in the United States in 1996–1997 would have stayed in the corresponding series in the subsequent year [11]. Studies of people who have not reached retirement age have found evidence of solid health care spending persistence. Hirth et al. (2015) found that medical expenses among the U.S. population under 65 show a strong and lasting pattern, particularly in the top 10% spending group [12]. There is a 34.4% chance that this high-expenditure group will still be in that category after 5 years, and their disease symptoms persist. Retired seniors also exhibit strong persistence in health care spending. Rettenmaier and Wang (2006) found significant persistence in Medicare reimbursements for the elderly in the U.S. Individuals who receive an extra dollar in Medicare reimbursements from the previous year may expect an increase of around $0.19 in reimbursements for the current year [13]. Figueroa et al. (2019) studied Medicare beneficiaries and confirmed that 28.1% of the 2012 high-spending group (the top 10% in each year) would have sustained high spending over the next two years. Similar findings are found in other developed regions [14]. Ku et al. (2015) show that the high-expenditure group, consisting of the top 10% spenders in Taiwan (China) from 2005 to 2009, has a 39% probability of remaining in the same group the following year [15]. Gastaldi-Ménager et al. (2016) found that 48% of France’s top 10% spending group in 2008 will continue to incur high medical expenditures in 2009 [16]. In terms of the healthcare spending concentration pattern, it was found that the top 5% spent about 50% of total healthcare expenditures from 1996 to 2012 in the United States (Cohen, 2016) [8]. In Taiwan (China), the top 10% spent about 55% of total expenditures from 2005 to 2009 (Ku et al., 2015) [15]. France’s top 10% spent around 62% of total expenditures in 2013 (Gastaldi-Ménageret al., 2016) [16].

There exists a lack of extensive research concerning children’s healthcare. One study conducted by Shenkman et al. (2007) looked at persistently high healthcare spending for children in the United States [6]. Given the differences in disease profiles between children and adults, evidence regarding adults may not be applicable to children. In addition, studies on the concentration and persistence of health spending mostly pertain to developed countries and regions. Only a limited number of empirical studies have been done regarding developing economies due to the availability of relevant data. The results of such studies in developing countries such as China may be different given differences in the medical systems [17]. Moreover, the time frame in most of these studies is relatively brief, the information they reveal regarding the dynamics of health expenditure is thus limited.

This paper fills a gap in the literature by examining the concentration and persistence of high health care spending in China, using a unique individual-level administrative claims dataset over a 5-year period. This is a unique study that investigates the concentration and persistence of health care expenditures for children in developing countries, based on long-term administrative claims data from a metropolitan city in China. The paper addresses three questions: (1) How much of the total children healthcare expenditures are consumed by the high-spending group? What are the key characteristics that distinguish high-cost patients from others? (2) What factors contribute to the emergence and persistence of high-cost patients? (3) Do the high-spending patients receive adequate protection from the medical system?

Here are three findings by using a linear probability model and logit regression. First, the top 10% of spenders consumed more than half of the total expenditures. The high-cost patients were mostly infants under 12 months or adolescents between 12 and 17 years old. Male children, children from poor families, and children diagnosed with a pediatric complex chronic condition (CCC) are more likely to become high-cost patients than others.

Second, age influenced the onset and continuity of high health care spending. Infants were more likely to become high spenders but less likely to stay as such. Conversely, the 6–11 and 12–17 age groups were more susceptible to both becoming and staying as high spenders. Pediatric CCCs and other serious conditions, such as leukemia, cardiac issues, renal disorders, and aplastic anemia, are significant predictors of entry and persistence in the high-cost group. Children from low-income families exhibited a tendency to become high spenders, but not necessarily to persist in that category.

Finally, Children from low-income families with high health expenditures also faced higher out-of-pocket expenditures than others. The findings from this research should serve as reminders to policymakers of the importance of risk protection for low-income families with high health care costs for children.

This study makes three contributions to the literature. First, it examines the persistence of children’s health expenditures in the largest developing economy. Previous studies on developed countries have shown that health expenditures of adults and especially the elderly are highly persistent [12, 15, 16, 18]. Is this also true for children in developing countries? This paper aims to answer this question and fill the research gap on developing countries. Second, this paper has an advantage over existing studies in that it uses data with a longer time span. Long-term data are crucial for studying the concentration and persistence of health care expenditures. Some diseases (such as malignant neoplasms) take a long time to cure, and short-term data may not capture their effects. Moreover, the range of diseases that can be treated and cured varies with advancements in technology. Persistence levels may change accordingly, and long-term data can reveal these trends.

Second, this paper contributes to the literature by presenting new evidence on the concentration of children’s health expenditure patterns using data from China. Previous studies on developed regions have shown that a small fraction of adults and children account for a large portion of health resources [8, 19–21], highlighting the need for and importance of strategic improvement of the healthcare system and care management. The findings from developing countries like China may inform local policymakers to adopt relevant policy suggestions.

Third, this study adds to the existing literature on risk protection for high-cost health care. In many Asian countries, out-of-pocket (OOP) payments are the main source of health care expenditures, which increases the risk of poor families facing catastrophic spending [7]. Previous research suggests that the level of protection, rather than the availability of insurance, is the crucial factor in preventing catastrophic spending, especially for low-income groups [22]. This study confirms that high-cost patients with low socioeconomic status also face higher OOP payments. This finding has implications for many low- and middle-income countries that have recently expanded or are planning to expand their public health insurance programs [23]. It highlights the need for adequate protection for low-income households with high health care expenditures.

## Data and methodology

This article analyzed inpatient claims data from the New Rural Cooperative Medical Insurance Scheme (NCMS) 2 in Sanming from 2010 to 2014^3^. Sanming is a modern city in China with a population of over 2 million. The data contained information on health care utilization, expenditures, and patient characteristics. Health care utilization data include admission and discharge dates, diagnoses, and health care facility levels. Expenditure data includes total expenditures, out-of-pocket (OOP) spending, prescription fees and medical tests fees. The data also reflected the secondary reimbursement from major illness insurance and other policy subsidies for poverty alleviation, such as those from the civil affairs department. The out-of-pocket (OOP) expenditure refers to the net amount remaining after subtracting all reimbursements and subsidies that the patient may receive. Patient information consists of date of birth, gender, and whether the patient belonged to a deprived family (*Dibao*). As with other administrative data, this dataset did not include educational records.

This dataset includes individuals aged 17 and younger, and excludes observations with negative or missing values for key variables (age, gender, and economic status). Based on the recommendations of Shenkman et al. (2007), total annual health care expenditures for recipients with more than one claim in a calendar year are computed by adding up all the expenditures in that year [6]. This method yields a total of 131,833 individuals with annual observations^4^. The recorded cost values are converted to 2014 RMB using the rural residents’ medical cost Consumer Price Index for the province where this city is located. Following Figueroa et al. (2019) [14], recipients whose annual cost was in the top 10% of spending are classified as high cost for each year.

To investigate the factors that contributed to the initiation and persistence of the high-spending group, this article first studied the odds of an individual being in the group with the top 10% of expenditures during any year and performed the following equation:

1 $$ {H}_{i,d,t}={\alpha }_{1}+{X}_{i,d,t}{\alpha }_{2}+{\theta }_{d}+{\mu }_{t}+{e}_{i,d,t}$$

Where $$ i$$ is individual, $$ d$$ county and $$ t$$ year. $$ {H}_{i,d,t}$$ is an indicator of individuals in the high-spending group; it equals 1 for a recipient in the group with the top 10% of expenditures during any calendar year and 0 otherwise. $$ {X}_{i,d,t}$$ is a vector of covariates, including the age and gender of individual $$ i$$, whether the individual belongs to a deprived family (*Dibao*)^5^, the level of health care institutions (primary/secondary/tertiary)^6^, and whether individual $$ i$$ has been hospitalized more than once during one calendar year (i.e., multiple readmissions).

$$ {X}_{i,d, t}$$ also includes several diagnoses/conditions to measure health status. Following Iizuka and Shigeoka (2019), one health indicator in our study is pediatric complex chronic conditions (CCCs) [3]. Appendix Table A1 shows the list and descriptive statistics of pediatric CCCs. Another health indicator is Ambulatory Care Sensitive Conditions (ACSCs), which are a set of preventable conditions that are common in children. The list of childhood ACSCs by Gadomski et al. (1998) is used [25]. Appendix Table A2 lists the ACSCs and their diagnostic codes according to the 10th revision of the International Classification of Diseases (ICD-10), and Table A3 shows the proportion of each ACSC in the sample. The third health indicator covers specific diagnoses, such as leukemia, cardiac conditions, renal disorders, or aplastic anemia.

$$ {\theta }_{d}$$ is the county fixed effect, and $$ {\mu }_{t}$$ denotes the year and month of the admission fixed effect. $$ {e}_{i,d,t}$$ is a random error term. We developed both a linear probability model and a logit regression model for Eq. (1).

This article also examined the likelihood of children in the top 10% of spenders in 2010 staying in this group in the following years until 2014. For example, to analyze the persistence of high spending from 2010 to 2011, this study first selected individuals with health expenditures in both years and identified those who were in the top 10% of spenders in both years as the persistent high-spending group. Then ran a regression of this binary variable on age, gender, diagnosis, economic status, and other covariates. This work followed the same procedure for the persistence of high spending from 2010 to 2012, 2010 to 2013, and 2010 to 2014. The estimation formula is:

2 $$ {Persistentlyhigh}_{i,d,t}={\alpha }_{3}+{X}_{i,d,t}{\alpha }_{4}+{\theta }_{d}+{\mu }_{t}+{e}_{i,d,t}$$

where $$ i$$ is individual, $$ d$$ county and $$ t$$ Year. $$ Persistentlyhigh$$ equals 1 for individuals who had health expenditures and were in the group with the top 10% of expenditures during 2010 and 2011, 2010 and 2012, 2010 and 2013, and 2010 and 2014. $$ {X}_{i,d,t}$$, $$ {\theta }_{d}$$, $$ {\mu }_{t}$$ and $$ {e}_{i,d,t}$$ share the same definition as used in Eq. (1). A linear probability model was developed for Eq. (2).

## Results

### Spending concentration

**Fig. 1:**
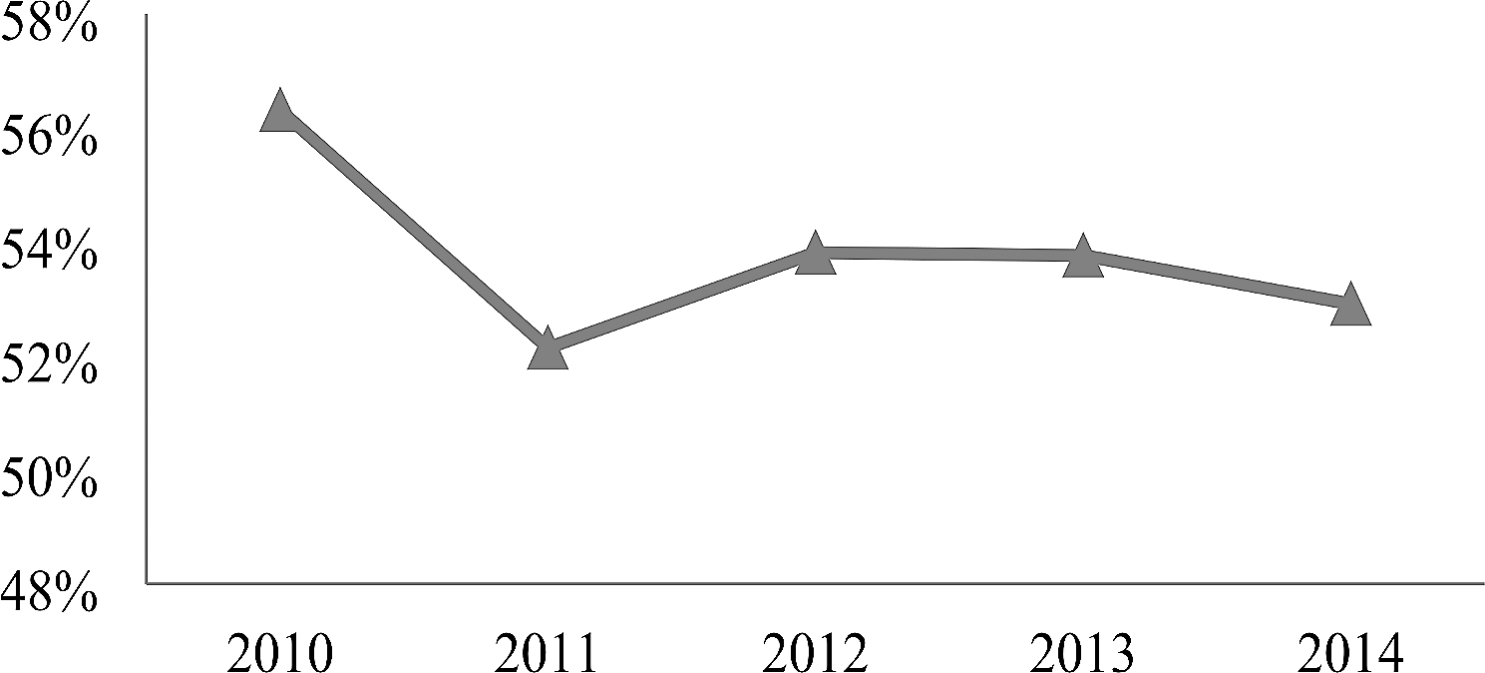
Concentration of children’s health Spending in China *Notes*: The data come from a unique individual level claim dataset in one metropolitan region, namely, Sanming city in Fujian Province in China with a 5-year observation period. This figure described the top 10% users spend how much percent of medical resources during each year from 2010 to 2014

Figure 1 illustrates the concentration of healthcare spending. More than 50% of the total spending came from individuals in the top 10% of spenders. This result suggests that children with the highest spending need more risk protection.

### Demographic and diagnostic characteristics of members of the high-spending group

Given the high concentration of health spending, it is important to identify the characteristics of patients who account for most of the spending, for the sake of risk protection and cost management. Table 1 shows the summary statistics for children in the high-spending group and the rest. This study classified individuals in the top 10% of spenders as the high-spending group. The data reveal significant differences between the high-spending group and the rest of the population. The high-spending group was more likely to include children under 12 months or between 12 and 17 years old, males over females, poor individuals (*Dibao*) 7, and those who received care in tertiary health facilities.

Table 1 shows that patients in the high-spending group tend to have more serious and chronic conditions than other patients. The likelihood of being diagnosed with a pediatric CCC was 8.9% for patients in the top 10% of spending, compared to only 0.8% for other patients. Among those who were admitted, 4.5% of the high-spending patients had leukemia, cardiac conditions, renal disorders, or aplastic anemia, which are severe diseases in children. Only 0.3% of the other patients had these conditions. The chance of being diagnosed with an ACSC was lower for high-cost patients than for low-cost patients (11.1% versus 37.2%). This is expected, as ACSCs are conditions that can be prevented by timely and effective primary care [25], implying that ACSC-related admissions may incur lower spending. At the same time, patients in the top 10% of spending were more likely to have multiple admissions in a calendar year.

**Table 1:** Characteristics of beneficiaries, by high-cost status, 2010-14

Variables	(1)	(2)
	High Spenders	Others
***Panel A. Demographic Characteristics***		
Age		
Younger than 12 months	0.321	0.178
	(0.467)	(0.382)
12 months to 5 years	0.283	0.496
	(0.451)	(0.500)
6 to 11 years	0.172	0.207
	(0.377)	(0.405)
12 to 17 years	0.224	0.120
	(0.417)	(0.325)
Mean age	5.526	4.678
	(5.894)	(4.640)
Male	0.676	0.643
	(0.468)	(0.479)
Belonged to a deprived family	0.019	0.015
	(0.138)	(0.121)
***Panel B. Diagnoses Characteristics***		
Any pediatric complex chronic conditions	0.089	0.008
	(0.285)	(0.088)
Neurologic and neuromuscular	0.008	0.000
	(0.090)	(0.020)
Cardiovascular	0.023	0.001
	(0.150)	(0.028)
Renal and urologic	0.002	0.000
	(0.047)	(0.006)
Gastrointestinal	0.007	0.000
	(0.085)	(0.018)
Hematologic or immunologic	0.004	0.001
	(0.066)	(0.024)
Metabolic	0.003	0.002
	(0.057)	(0.043)
Other congenital or Genetic defect	0.003	0.000
	(0.056)	(0.011)
Malignancy	0.016	0.000
	(0.126)	(0.021)
Premature and neonatal	0.021	0.003
	(0.144)	(0.057)
Any ambulatory care sensitive conditions	0.111	0.372
	(0.314)	(0.483)
Any leukemia, cardiac, kidney or	0.045	0.003
aplastic anemia conditions	(0.206)	(0.054)
Hospitalized for more than one time within one year	0.419	0.174
	(0.493)	(0.379)
Primary Institutions	0.033	0.375
	(0.179)	(0.484)
Secondary Institutions	0.304	0.496
	(0.460)	(0.500)
Tertiary Institutions	0.663	0.129
	(0.473)	(0.336)
Observations	13,181	118,652

*Notes*: The data come from a unique individual level claim dataset in one metropolitan region, namely, Sanming city in Fujian Province in China with a 5-year observation period. We merge the top 10% spending for each calendar year as the high spenders. Standard deviations are in brackets

Appendix table A4 shows the top 10 diagnostic categories for the high-spending group, which made up 85.5% of total admissions. The results suggest that acute diseases were prevalent among children in the region and contributed to high health spending. Perinatal conditions were also very common, reflecting the specific nature of children’s diseases. Infectious diseases are another frequent cause of illness among children in China.

### Spending patterns by high-cost status

**Fig. 2:**
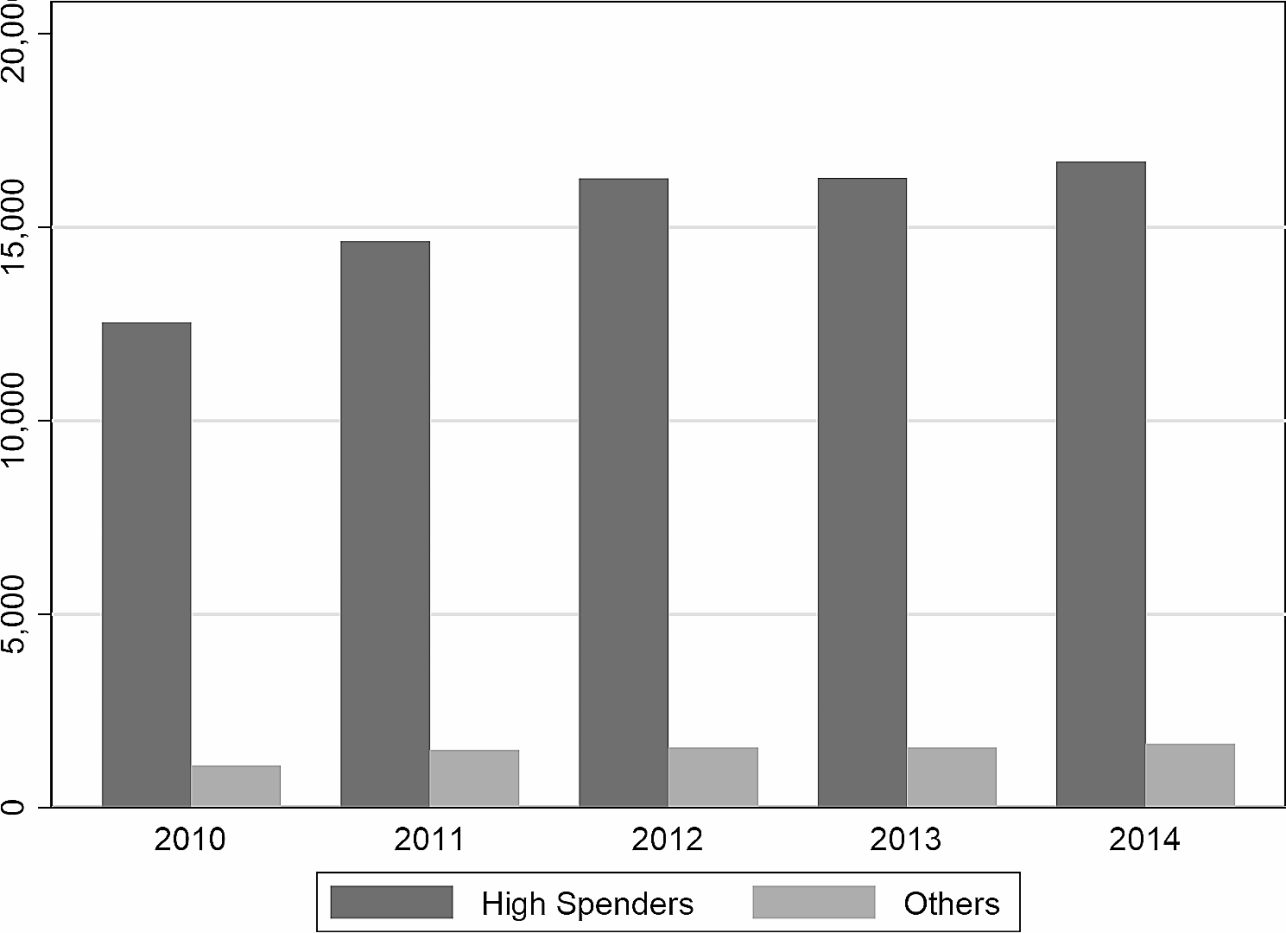
Average per person per year total spending for beneficiaries, by high-cost status, 2010–14 *Notes*: The data come from a unique individual level claim dataset in one metropolitan region, namely, Sanming city in Fujian Province in China with a 5-year observation period. Recipients whose annual cost was in the top 10% of spending were categorized as high cost for each individual year

This study compared the spending patterns of patients in the high-spending group and the rest of the patients. Fig. 2 shows that, on average, high-spending patients spent 12,543 RMB ($1,896) in the first year, while the other patients spent only 1,088 RMB ($164). In the following years, the high-spending patients increased their spending slightly more than in the first year. The other patients also spent more in the subsequent years, but the increase was small.

Table 2 shows that high-cost patients spent more on all categories of spending, especially on drugs and tests. Their drug spending (7,832 RMB) was nine times higher than that of low-cost patients (830 RMB). Their test spending (5,332 RMB) was almost eleven times higher than that of low-cost patients (494 RMB). They were also more than five times more likely to undergo surgical procedures (0.43 versus 0.08). Their surgery spending (5,120 RMB) was over ten times higher than that of low-cost patients (484 RMB).

**Table 2:** Total mean yearly spending, by high-cost status, 2010–14

Variables	(1)	(2)
	High spenders	Others
Total Spending (RMB)	15,566	1,504
Drugs (RMB)	7,832	830
Tests (RMB)	5,332	494
Ever do Surgery	0.43	0.08
Surgery fees (RMB)	5,120	484

*Notes*: The data comes from a unique individual level claim dataset in one metropolitan region, namely, Sanming city in Fujian Province in China with a 5-year observation period. The average exchange rate between RMB and U.S. dollars is 6.3754 during 2010 and 2014

### Odds of being in the Group with the top 10% of expenditures in any year

This article analyzed the likelihood of a child belonging to the top 10% of spenders in any of the five years of the observation period. Table 3 reports the results. Columns (1) and (2) show the estimates of linear probability and logit regression, respectively. Demographic characteristics are significantly associated with the likelihood of being a high-cost patient (i.e., in the top 10% of health care spending). Infants are more likely to be high-spending targets than children aged 1 to 5 years. The marginal effect is 4.7% for both the linear probability model and the logit regression. Children aged 6 to 11 years and 12 to 17 years are also more likely to be high-cost patients than those aged 1 to 5 years.

Young children have a higher chance of being high-spending targets, which may be related to their risk of prematurity [26] and congenital anomaly surgeries [27]. Adolescents can also have high spending, which may be related to unhealthy lifestyles and risky behaviors as well as emerging mental health issues [28].

Males are 0.5% more likely to join the top 10% of health care spenders than females, a finding that differs from Shenkman et al. (2007) [6]. This observation could be linked to the cultural preference for boys in China, suggesting a tendency to not only favor male children but also to indulge and pamper them more. Children from deprived families tend to have a higher chance of being in the top 10% of health care spenders. This finding indicates that enabling characteristics are important for predicting health spending.

Patients with any pediatric CCCs were 25.9% more likely to be high-cost beneficiaries than patients without these conditions. Patients with other serious conditions were 23.9% more likely to be in the top 10% of spenders than other patients. This finding shows that CCCs and other serious conditions are very important indicators of high health spending. Patients with ACSC hospitalizations have a lower chance of joining the top 10% of spenders. This finding is expected, as ACSCs are conditions that can be prevented by timely and effective primary care [25].

Patients who were hospitalized in secondary and tertiary facilities were more likely to be in the top 10% of spenders than patients who were admitted to primary facilities. A patient’s admission to a hospital more than once in a calendar year was a significant positive indicator. This finding shows that the number of hospital admissions is highly useful for predicting high spending.

**Table 3:** Odds of being in the top 10% of Spenders in any year

Variables	(1)	(2)
	Linear Probability Model	Logit
Age		
Younger than 12 months	0.047***	0.047***
	(0.002)	(0.002)
6 to 11 years	0.038***	0.041***
	(0.002)	(0.002)
12 to 17 years	0.107***	0.096***
	(0.002)	(0.002)
Male	0.005***	0.005***
	(0.001)	(0.001)
Belonged to a deprived family	0.010*	0.006
	(0.006)	(0.005)
Any pediatric complex chronic conditions	0.259***	0.086***
	(0.006)	(0.004)
Any ambulatory care sensitive conditions	-0.054***	-0.074***
	(0.002)	(0.002)
Any leukemia, cardiac, kidney or	0.239***	0.074***
aplastic anemia conditions	(0.009)	(0.005)
Hospitalized for more than one time within one year	0.121***	0.095***
	(0.002)	(0.001)
Secondary Institutions	0.052***	0.123***
	(0.002)	(0.003)
Tertiary Institutions	0.327***	0.258***
	(0.002)	(0.003)
Observations	131,833	131,833
R-squared	0.255	

*Note*: The data come from a unique individual level claim dataset in one metropolitan region, namely, Sanming city in Fujian Province in China with a 5-year observation period. Robust standard errors are in brackets. *** *p* < 0.01, ** *p* < 0.05, * *p* < 0.1. Logit regression reports the marginal effect at the mean. “12 months to 5 years” is the reference age group. “Primary Institutions” is the reference institution group. Year and month fixed effect and county fixed effect are included in all regressions

### Odds that children in the group with the top 10% of expenditures in 2010 remained in this group in 2011, 2012, 2013 and 2014

This study analyzed the persistence of spending by examining the likelihood of a child staying in the top 10% of spenders in 2011, 2012, 2013, and 2014, based on their spending rank in 2010. Table 4 shows the results of our model. Columns (1)-(4) show the likelihood of high-cost patients in 2010 remaining in that group in 2011, spanning through 2012, 2013, and until 2014, respectively. For Column (1), this article selected patients in the top 10% of spenders in both 2010 and 2011 as the persistent high-spending group. The same definition of persistent high-cost patients is applied for Columns (2)-(4).

Demographic characteristics are significant predictors of the chance of staying in the top 10% of health care spenders. Children aged 6 to 11 years and 12 to 17 years are more likely to stay in this high-spending group than those aged 1 to 5 years from 2010 to 2013, which agrees with Shenkman et al. (2007) [6]. Infants are less likely than children aged 12 months to 5 years to stay in the top 10% of spenders, but this only holds for 2010 to 2011. This differs from Shenkman et al. (2007) [6], which suggests that infants in China are not likely to remain high-spending for a long time, although they have a higher chance of joining the high-spending group.

The coefficients of age cohorts for the analysis from 2010 to 2014 are not significant. This may be because of the low number of patients observed over a longer period; that is, very few children had positive health care spending in all five years. The data show that only 635 children had persistent spending in this period, making up only 0.5% of the sample.

**Table 4:** Odds of remaining in the top 10% of Spenders in descending years, given presence in the top 10% of Spenders in 2010

Variables	(1)	(2)	(3)	(4)
	From 2010 to 2011	From 2010 to 2012	From 2010 to 2013	From 2010 to 2014
Age				
Younger than 12 months	-0.019***	-0.003	-0.013	-0.010
	(0.006)	(0.007)	(0.011)	(0.018)
6 to 11 years	0.028***	0.012***	0.015**	0.011
	(0.005)	(0.004)	(0.006)	(0.008)
12 to 17 years	0.070***	0.061***	0.038***	-0.028
	(0.006)	(0.007)	(0.013)	(0.018)
Male	-0.005	0.006*	0.012**	0.009
	(0.004)	(0.003)	(0.005)	(0.007)
Belonged to a deprived	0.003	-0.035**	-0.033	0.017
Family (*Dibao*)	(0.015)	(0.014)	(0.022)	(0.026)
Hospitalized for more than	0.015***	0.004	0.006	0.003
one time within one year	(0.004)	(0.003)	(0.005)	(0.006)
Any pediatric complex	0.247***	0.296***	0.350***	0.275***
chronic conditions	(0.017)	(0.017)	(0.019)	(0.020)
Any ambulatory care	-0.016***	-0.006**	-0.002	-0.004
sensitive conditions	(0.004)	(0.003)	(0.005)	(0.006)
Any leukemia, cardiac, kidney	0.180***	0.098***	0.252***	
or aplastic anemia conditions	(0.019)	(0.020)	(0.025)	
Secondary Institutions	0.026***	0.010**	-0.002	0.011
	(0.004)	(0.004)	(0.006)	(0.008)
Tertiary Institutions	0.105***	0.018***	0.027***	-0.019*
	(0.006)	(0.006)	(0.008)	(0.011)
Observations	6,872	3,072	1,348	635
R-squared	0.165	0.182	0.420	0.318

*Notes*: Robust standard errors are in brackets; *** *p* < 0.01, ** *p* < 0.05, * *p* < 0.1. “12 months to 5 years” is the reference age group. “Primary Institutions” is the reference institution group. Year and month fixed effect and county fixed effect are included in all regressions

Children with more severe health conditions have a higher chance of staying in the top 10% of health care spenders. Gender, economic status, and multiple readmissions in a year are not significant predictors of the chance of staying in the top 10% of health care spenders^8^. Hospitalizations in secondary and tertiary facilities are significantly associated with the likelihood of remaining in the high-spending group.

To summarize, infants are more likely to join the high-spending group but less likely to stay in it over time. On the other hand, children aged 6 to 11 years and 12 to 17 years are more likely to both join and stay in the high-spending group. Pediatric CCCs and other serious conditions are strong predictors of being and staying in the high-cost group. Lower economic status is an indicator of entering the high-spending group but not strongly correlated with high-spending persistence pattern. This suggests that children from low-income families are more probable to have high medical spendings and also tight budget constraints.

## Conclusions and discussions

This paper uses a unique individual-level, 5-year claim dataset from a metropolitan city in China and provides strong evidence on the long-term concentration and persistence of children’s health care spending. Our research aims to understand multiple factors contributing to persistent high spending among children and provide empirical evidence of disproportionate spendings among children patients.

We found that more than 50% of the total spending came from the top 10% of children. The high-spending group was more likely to include children under 12 months or between 12 and 17 years old, boys, low-income families, and those diagnosed with a pediatric complex chronic condition. This study’s findings on the health status of high-spending children are in line with the literature on the health spending of children with special needs. For example, Newacheck & Kim (2005) found that 20% of children with chronic conditions in the United States accounted for 80% of the pediatric health care spending [29].

Specific enabling and need characteristics are related to the persistence of high health care spending. This agrees with the results from developed countries. It shows that these characteristics can help identify children who need care management and coordination, which are common practices in developed regions but not in developing countries.

Age is a significant factor in the onset and continuity of high health spending. Infants are more likely to start high spending but less likely to stay in it over time. This may be due to factors such as preterm birth [30], meconium aspiration syndrome [31], and congenital anomaly surgeries [32]. These conditions can be treated with acute, timely, and effective care and can lower the risk of hospitalization. Adolescents can also have high spending, which may be related to their unhealthy lifestyles and risky behaviors as well as their emerging mental health issues [28].

The health status of young patients is a key determinant of both the onset and persistence of high medical costs. Young patients with pediatric CCCs or other serious conditions are more likely to belong to the top 10% of medical cost users. Therefore, developing countries need to implement care-management and care-coordination to improve the outcomes for this certain type of children.

Low economic status can be used to predict the probability of becoming high-spending patients, but not staying in that pattern. However, this does not mean that low-income households lack a persistent need for high medical costs, which may be constrained by their budget. Since the average reimbursement rate of the NCMS is only slightly above 50%, families may forgo medical treatment when they cannot afford the remaining 50%, and thus they will not appear in our spending data.

**Fig. 3:**
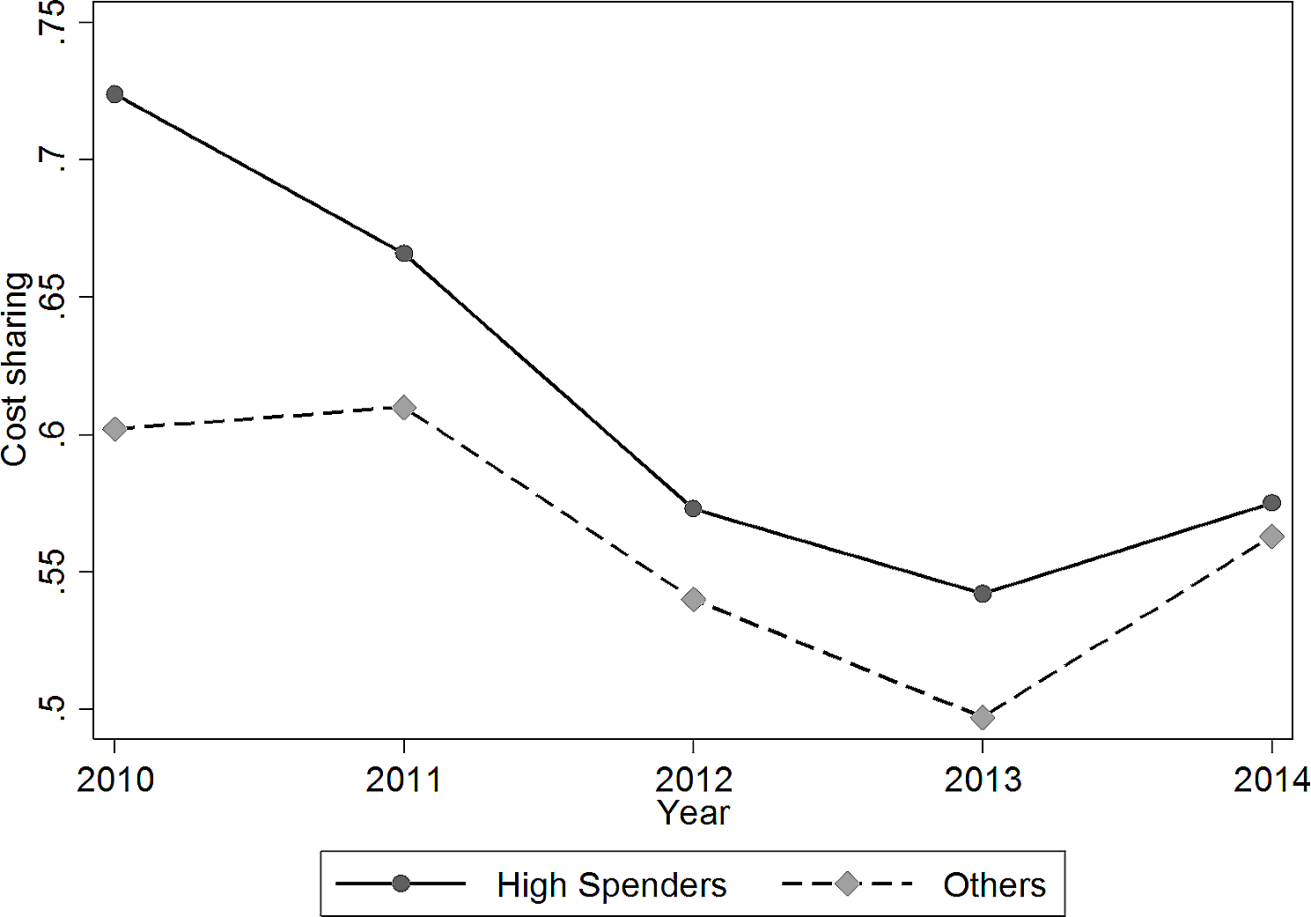
Average individual cost share for the top 10% and the other 90% of spenders *Notes*: The data come from a unique individual level claim dataset in one metropolitan region, namely, Sanming city in Fujian Province in China with a 5-year observation period

Protecting patients against catastrophic spending is essential given the persistence of high health expenditures. Figure 3 shows that high-cost patients had a higher cost share on average. Although it decreased over time, the cost share remained over 50%. However, we cannot infer that high spenders were poorly protected because household income was not reported. Thankfully, the data provided information on whether the patient belonged to a poor household (*Dibao*), and this study examined the protection level of poor households in the high-cost group based on this condition. Specifically, the data indicated that the cost share of poor households in the high-cost group (i.e., 58%) was higher than the average level of the entire population (i.e., 56%). Moreover, the average health expenditure of poor households in the high-cost group was 15,055 RMB (i.e., $2,375), slightly lower than that of others (15,578 RMB, which is $2,528) but much higher than the average disposable income of the entire population, let alone the income of poor households^9^. This suggests that policymakers should offer more insurance benefits and medical support for poor households with high costs.

In addition to the health expenditure characteristics examined in this paper, another issue that requires attention is the low participation rate of young patients in the medical insurance program. Children exhibit lower enrollment rates than adults [33, 34], especially when lacking hukou, being urban migrants, or being preschoolers [33]. There are several possible reasons. First, parents have limited access to the policy information on enrollment, and grassroots organizations have no incentive to promote the policy to new families. Second, some parents may exclude their children from the scheme due to budget constraints or unwillingness to pay as the premium increases. For example, the personal payment of the new rural cooperative medical scheme has risen from 10 RMB to 180 RMB. Third, the social medical insurance policy applies the same reimbursement standard to children and adults. With the primary goal being the “protection against serious illnesses,” the policy does not provide reimbursement for preventive healthcare expenses that children typically require, nor does it cover outpatient expenditures related to minor illnesses that children are more susceptible to. This lack of coverage may lead to frustration among parents who have expectations of comprehensive policy benefits. Lastly, the migration of children from rural to urban areas leads to a situation where urban migrant children encounter a lack of opportunities to participate in insurance programs. Migrated children can join the new rural cooperative medical scheme when they are in rural areas, but they may not qualify for the urban residents’ medical insurance due to the lack of urban hukou. It is recommended that the policy should be improved in the following aspects. First, the government should lower the deductible standard of children’s insurance reimbursement and increase the benefit rate. Children’s medical reimbursement currently follows the same deductible standard as adults in China. However, the low drug dosages for children, coupled with lower treatment costs, contribute to a diminished insurance benefit rate for pediatric care. Second, the government should remove the hukou restriction and allow migrated children to participate in the basic medical insurance of their destination location. Third, encourage grassroots organizations to enhance policy publicity and increase the availability of information on children’s enrollment for parents.

It is important to note that, due to data limitations, this paper only examines the health expenditure characteristics of the NCMS. However, the merged scheme of the NCMS and the URBMI in 2016 also significantly impacted the dynamic of health expenditure. The merged scheme increased the use and spending of medical services for rural residents in China considerably [35–37], especially for the elderly population [38]. Feng et al. (2022) estimated the optimal medical insurance reimbursement level that strikes a balance between efficiency and equity [36]. Future research is needed to assess the effects of the merged scheme and provide evidence for policy improvement.

This paper presents interesting results regarding health expenditures’ skewed distribution and persistence, but it also faces some limitations. First, this research relies on a relatively limited sample. The data comes from individual-level reimbursement records of one city. These results may lack generality for the whole country. However, the limited dataset is a common problem in research along with the rising availability of administrative data, especially in developing countries. We compared the selected city with other Chinese cities to demonstrate its representativeness. As shown in Appendix Table A5, the selected city is comparable to the median city in terms of government revenues, number of hospitals, and other characteristics. This implies that our findings could be applicable and generalizable when it comes to the persistence and concentration of children’s health expenditures in China.

Although the sample data may be somewhat outdated, the findings of this study remain relevant today as they are comparable with those observed in developed areas. For instance, this study reveals that age and health status are significant factors affecting the continuity of medical expenditure, aligning with similar findings from Shenkman et al. (2007) in the U.S [6]., among other factors. In general, the disease spectrum of children is relatively simple and stable compared to that of adults. Given a series of sustained public health interventions in China since 1949, the disease spectrum of children during our sample period has been very close to that of developed countries (Chinese Health Statistics Yearbook, 2015), and has evolved without major changes to the present (Chinese Health Statistics Yearbook, 2023). In other words, the core factors affecting the sustainability of healthcare expenditures have not changed substantially. Thus, there is reason to believe that the study’s conclusions are still relevant now. Moreover, China’s health insurance policy has remained largely unchanged up to now (Chinese Health Statistics Yearbook, 2023). Therefore, this study’s results are still applicable in the present context. If future data is available, more studies will follow.

Second, we could not estimate the data attrition due to death since this research had no records of deaths. Despite these limitations, this research offers compelling evidence regarding the health spending persistence, although the possibility of death may weaken this evidence. Therefore, we should view the persistence reported by our paper as a lower boundary.

This study has important policy implications for disease management and risk protection, especially for households facing high medical costs due to a child’s illness. While studies about adult health spending often focus on cost control [e.g., 39], this research emphasizes the significance of health investments during childhood, which can impact long-term health status and socioeconomic outcomes. Additionally, the price insensitivity of children’s utilization of inpatient care suggests that beyond cost containment, policymakers should consider risk protection measures [4]. This research serves as a reminder of the necessity to prioritize disease management and risk protection for economically vulnerable households facing elevated medical costs for children.

### Electronic supplementary material

Below is the link to the electronic supplementary material.

Supplementary Material 1

## Data Availability

The data that support the findings of this study are available from the Sanming Medical Insurance Bureau, Fujian province of China. Restrictions apply to the availability of these data, which were used under license for this study. Data are available from the corresponding author with the permission of the Sanming Medical Insurance Bureau.

## Footnotes

1 China has achieved the goal of providing universal health coverage; more than 1.33 billion people, i.e., 97.5% of the Chinese population, are covered by the public health insurance program (Chinese Health Statistics Yearbook, 2015).

2 China has achieved universal health coverage, with more than 1.33 billion people, or 97.5% of its population, enrolled in the public health insurance program (Chinese Health Statistics Yearbook, 2015). The program consists of three schemes: the Urban Employee Basic Medical Insurance (UEBMI) for the urban employed, the New Rural Cooperative Medical Insurance Scheme (NCMS) for rural residents, and the Urban Resident Basic Medical Insurance (URBMI) for urban residents without formal employment, especially the elderly and children. The government also offers medical assistance to those with low socioeconomic status. The appendix contains a detailed introduction to the background of China’s medical service and insurance system for interested readers.

3 This metropolitan city in China has both rural and urban registered populations.

4 For the analysis of disease/condition, we employ the first claim records instead of the total spending calculation. This approach yields 169,633 observations.

5 Rural *Dibao* is a minimum living standard guarantee program in China. The poverty line (Dibao line) varies across regions. Village leaders identify and screen potential beneficiaries in practice (Kakwani et al., 2019) [24].

6 China has minimal gatekeepers to hospital services (no referral system), and patients have direct access to specialists. Deductibles, copayments, and reimbursement caps improve the utilization efficiency of medical resources, and they vary based on three tiers of medical facilities: primary (township/community health centers), secondary and tertiary facilities. Tertiary facilities have the highest deductibles and copayments, followed by secondary and primary facilities.

7 Appendix Figure A1 further illustrates the mean expenditure for every age category in the top 10% of expenditures.

8 It is noteworthy that although the gender effects in the regression from 2010 to 2012 are double those of the regression from 2010 to 2013, the former’s standard error is also almost double that of the latter. This casts doubt on the statistical difference between the gender effects in the two sets of models. Therefore, we verified it using the seemingly unrelated regression (SUR) model. The results indicate that the corresponding p-value is 0.1649, implying that there is no significant difference between the gender effects in the two sets of models.

9 The mean expenditure for deprived families was 15,055 RMB (i.e., $2,375). Meanwhile, the income per person for rural residents in the sample city was 8,765 RMB (i.e., $1,382), and deprived families earned even less.

## Abbreviations

OOP: Out-of-pocket
NCMS: New Rural Cooperative Medical Insurance Scheme
UEBMI: The Urban Employee Basic Medical Insurance
URBMI: Urban Resident Basic Medical Insurance
CCCs: Pediatric complex chronic conditions
ACSCs: Ambulatory Care Sensitive Conditions
