# Supplementary material for: Characteristics and spending patterns of high-cost child patients: findings from Fujian in China
Source: BMC Public Health. 2024 May 15;24:1284. doi: 10.1186/s12889-024-18246-x (PMC11094993; doi:10.1186/s12889-024-18246-x)
Supplement: Supplementary file 1 — Supplementary Material 1 [file 12889_2024_18246_MOESM1_ESM.docx]

**Appendix A. Supplementary figures and tables**

**Figure A1 Average Spending of the Top 10% of Users by Age Group**

*Notes*: The data come from a unique individual level claim dataset in one metropolitan region, namely, Sanming city in Fujian Province in China with a 5-year observation period.

Figure A1 demonstrates average spending for each age group among those in the top 10% of expenditures. Average spending for each age cohort is U shaped. This finding is consistent with those of studies conducted in developed regions. Shenkman et al. (1996) and Shenkman et al. (2007) show that very young children and adolescents exhibit the highest health care use and that school-age children exhibit the lowest health care use in the United States. There are several reasons for high spending among infants, such as stays in a neonatal intensive care unit (NICU) related to preterm birth (Blencowe et al., 2012), meconium aspiration syndrome (Kalra et al., 2020), surgical repairs of congenital anomalies (Kaltman et al., 2006), and other factors. Health spending among adolescents can be high due to the fact that adolescents engage in risk-taking behaviors and have other special health care needs (Youngblade et al., 2002).

**Table A1 List of pediatric complex chronic conditions (CCCs)**

| CCC categories | Mean | Number | *Share* |
| --- | --- | --- | --- |
| Neurologic and neuromuscular | 0.002 | 311 | *9.6%* |
| Cardiovascular | 0.003 | 472 | *14.6%* |
| Respiratory | 0.000 | 17 | *0.5%* |
| Renal and urologic | 0.000 | 79 | *2.4%* |
| Gastrointestinal | 0.001 | 185 | *5.7%* |
| Hematologic or immunologic | 0.002 | 273 | *8.4%* |
| Metabolic | 0.002 | 305 | *9.4%* |
| Other congenital or Genetic defect | 0.000 | 71 | *2.2%* |
| Malignancy | 0.005 | 801 | *24.7%* |
| Premature and neonatal | 0.004 | 723 | *22.3%* |
| Technology dependence | 0.000 | 17 | *0.5%* |
| Transplantation | 0.000 | 3 | *0.1%* |
| Any CCC | 0.019 | 3240 | *100%* |

*Notes*: The data comes from a unique individual level claim dataset in one metropolitan region, namely, Sanming city in Fujian Province in China with a 5-year observation period.

CCCs are defined by Feudtner et al. (2000, 2014) as “any medical condition that can be reasonably expected to last at least 12 months (unless death intervenes) and to involve either several different organ systems or 1 organ system severely enough to require specialty pediatric care and probably some period of hospitalization in a tertiary care center”.

See Feudtner et al. (2014) for corresponding ICD10, as well as Feudtner et al. (2000) for original list in ICD9.

References: Feudtner et al. (2014) “Pediatric complex chronic conditions classification system version 2: updated for ICD-10 and complex medical technology dependence and transplantation.” *BMC Pediatrics* 14:199. Feudtner et al. (2000) “Pediatric Deaths Attributable to Complex Chronic Conditions: A Population-Based Study of Washington State, 1980–1997” *Pediatrics* 106: 205–209.

Table A1 shows a total of 1.9% of children were diagnosed with one of the CCCs. Among the list, the top three are malignancy (24.7%), premature and neonatal conditions (22.3%) and cardiovascular conditions (14.6%), which make up over 60% of total CCCs. Our results are somewhat different from those of Iizuka and Shigeoka (2019), where the top three on the CCCs list are cardiovascular conditions, malignancy and metabolic conditions.

**Table A2 List of Ambulatory Care Sensitive Conditions (ACSCs)**

| ACSC categories | International Classification of Diseases (ICD-10) |
| --- | --- |
| Congenital syphilis | A50.0-A50.9 |
| Immunization preventable conditions | A35, A36, A37, A80, G00 |
| Grand mal status and other epileptic convulsions | G40, G41 |
| Convulsions "A" & "B" | R56 |
| Severe ENT infections | H66, H67, J02, J03, J06, J31.2 |
| Bacterial pneumonia | J13, J14, J15.3, J15.4, J15.7, J15.9, J16.8, J18, J18.1 |
| Asthma | J45, J46 |
| Tuberculosis | A15, A16, A17, A18, A19 |
| Cellulitis | L03 L04 L08.0 L08.8 L08.9 L88 L98.0 |
| Diabetes "A", "B", "C" | E10.0-E10.8, E11.0-E11.8, E12.0-E12.8, E13.0-E13.8, E14.0-E14.8 |
| Hypoglycemia | E16.2 |
| Gastroenteritis | K52.2, K52.8, K52.9 |
| Kidney/urinary infection | N10, N11, N12, N13.6 |
| Dehydration-volume depletion | E86 |
| Iron deficiency anemia | D50.1, D50.8, D50.9 |
| Nutritional deficiencies | E40, E41, E42, E43, E55.0, E64.3 |
| Failure to thrive | R629 |
| Any ACSC |  |

*Notes*: References: Gadomski, Anne, Paul Jenkins, and Melissa Nichols. (1998) “Impact of a Medicaid primary care provider and preventive care on pediatric hospitalization.” Pediatrics 101(3).

**Table A3 Summary Statistics of** **Ambulatory Care Sensitive Conditions (ACSCs)**

|  | Cond. On visit | Number | *Share* |
| --- | --- | --- | --- |
|  | (N=169,633) |  |  |
| ACSC categories | Mean |  |  |
| Congenital syphilis | 0.000 | 0 | *0.0%* |
| Immunization preventable conditions | 0.000 | 40 | *0.1%* |
| Grand mal status and other epileptic convulsions | 0.002 | 303 | *0.5%* |
| Convulsions "A" & "B" | 0.005 | 781 | *1.3%* |
| Severe ENT infections | 0.18 | 30558 | *50.4%* |
| Bacterial pneumonia | 0.125 | 21220 | *35.0%* |
| Asthma | 0.005 | 932 | *1.5%* |
| Tuberculosis | 0.001 | 102 | *0.2%* |
| Cellulitis | 0.003 | 518 | *0.9%* |
| Diabetes "A", "B", "C" | 0.000 | 0 | *0.0%* |
| Hypoglycemia | 0.000 | 18 | *0.0%* |
| Gastroenteritis | 0.036 | 6149 | *10.1%* |
| Kidney/urinary infection | 0.000 | 23 | *0.0%* |
| Dehydration-volume depletion | 0.000 | 2 | *0.0%* |
| Iron deficiency anemia | 0.000 | 26 | *0.0%* |
| Nutritional deficiencies | 0.000 | 10 | *0.0%* |
| Failure to thrive | 0.000 | 0 | *0.0%* |
| Any ACSC | 0.358 | 60,682 | *100%* |

*Notes*: The data come from a unique individual level claim dataset in one metropolitan region, namely, Sanming city in Fujian Province in China with a 5-year observation period.

References: Gadomski, Anne, Paul Jenkins, and Melissa Nichols. (1998) “Impact of a Medicaid primary care provider and preventive care on pediatric hospitalization.” Pediatrics 101(3).

Table A3 shows as much as 35.8% of the hospitalizations are due to ACSCs. This verifies the acute nature of children’s diseases. Our results are quite similar to those of Iizuka and Shigeoka (2019). Among the list of 17 ACSCs, severe ear, nose, and throat (ENT) infections (50.4%) and bacterial pneumonia (35%) account for nearly 90% of total ACSCs. This is slightly different from the results of Iizuka and Shigeoka (2019), where asthma ranked second.

**Table A4 List of the top 10 diagnostic categories for the group with the top 10% of expenditures**

|  | (1) | (2) |
| --- | --- | --- |
| Categories of Diagnose | *Ratio (%)* | International Classification of Diseases (ICD-10) |
| Diseases of the respiratory system | *18.6* | J00-J99 |
| Certain conditions originating in the perinatal period | *17.2* | P00-P96 |
| Injury, poisoning and certain other consequences of external causes | *11.9* | S00-T98 |
| Diseases of the digestive system | *10.2* | K00-K93 |
| Congenital malformations, deformations and chromosomal abnormalities | *7.5* | Q00-Q99 |
| External causes of morbidity | *6.6* | V01-Y98 |
| Certain infectious and parasitic diseases | *4.7* | A00-B99 |
| Diseases of the genitourinary system | *3.1* | N00-N99 |
| Neoplasms | *3.1* | C00-D48 |
| [Diseases of the nervous system](https://icd-codes.com/icd10cm/G00-G99) | *2.6* | G00-G99 |
| Total | *85.5* |  |

*Notes*: The data come from a unique individual level claim dataset in one metropolitan region, namely, Sanming city in Fujian Province in China with a 5-year observation period.

Table A4 provides the list of the top 10 diagnostic categories for the group with the top 10% of expenditures. This group accounts for 85.5% of total admissions. With respect to their diagnoses, diseases of the respiratory system account for 18.6% of total observations. Injury, poisoning and certain other consequences of external causes, diseases of the digestive system, and external causes of morbidity account for 11.9%, 10.2% and 6.6% of the total, respectively. These results indicate that children in the region under study mostly suffer from acute diseases and that these diagnoses play a large role in high health expenditures. Congenital malformations, deformations and chromosomal abnormalities also make great contributions, accounting for 7.5% of total diagnoses. Certain conditions originating during the perinatal period are very common (17.2%), indicating the unique nature of children’s diseases. Neoplasms account for 3.1% of total diagnoses, implying that children also suffer from those serious conditions. Diseases of the genitourinary system and diseases of the nervous system account for 3.1% and 2.6% of the total, respectively. Certain infectious and parasitic diseases account for 4.7% of the total, indicating that children in China also suffer from infectious disease.

**Table A5 Statistics for the sample city and all cities between 2010 and 2014 in China**

|  | Government revenue  (10k RMB) | Total population  (10k) | Per capita savings balance (RMB) | Per capita disposable income for urban residents (RMB) | Per capita disposable income for rural residents (RMB) | Number of primary schools | Internet users (household) | Number of hospitals | Number of hospital beds | Number of physicians |
| --- | --- | --- | --- | --- | --- | --- | --- | --- | --- | --- |
| Sample cities | 744759 | 277 | 21178 | 22031 | 8765 | 253 | 413904 | 161 | 11120 | 4517 |
| Percentiles for all cities in China | | |  |  |  |  |  |  |  |  |
| p5 | 172982 | 109 | 7739 | 13980 | 4210 | 95 | 96000 | 53 | 4236 | 2004 |
| p10 | 240339 | 144 | 9791 | 14877 | 4965 | 148 | 130000 | 72 | 5619 | 2584 |
| p25 | 427293 | 241 | 13868 | 16936 | 6231 | 287 | 220168 | 111 | 8842 | 4108 |
| p50 | 767556 | 375 | 20611 | 19726 | 7908 | 537 | 379921 | 168 | 13430 | 6569 |
| p75 | 1486523 | 582 | 36094 | 23456 | 10002 | 1007 | 660000 | 264 | 20955 | 10125 |
| p90 | 3407132 | 794 | 61890 | 29112 | 12685 | 1480 | 1477477 | 380 | 32597 | 17772 |
| p95 | 6264618 | 967 | 88581 | 33140 | 14928 | 1868 | 2178947 | 550 | 43355 | 22657 |

*Notes*: The data come from China City Statistical Yearbook, 2011-2015.

**Appendix B. Institutional framework of the Chinese health care system**

China has accomplished the goal of providing universal health coverage; more than 1.33 billion people, i.e., 97.5% of the population of China, receive coverage from the public health insurance program (Chinese Health Statistics Yearbook, 2015). The Urban Employee Basic Medical Insurance (UEBMI) is intended for the urban employed population, the New Rural Cooperative Medical Insurance Scheme (NCMS) is aimed at rural residents, and the Urban Resident Basic Medical Insurance (URBMI) targets urban residents who lack formal employment, especially elderly individuals and children. The government provides medical assistance in addition to public health insurance for individuals with lower socioeconomic status.

In this paper, we focused on the NCMS. The NCMS is financed by low individual contributions and high government subsidies. In 2011, for example, the average financing was 246 RMB (38 USD) per person, of which 84% was from government subsidies. The NCMS focuses its coverage on inpatient care. Following the broad guidelines issued by the central government, the NCMS is administered at the county level and varies across counties with respect to scheme features. All counties cover inpatient care, and the bulk of NCMS funds are used for inpatient expenses.

There are minimal gatekeepers to hospital services (no referral system), and patients can obtain access to specialists directly. To improve the utilization efficiency of health care resources, deductibles, copayments, and reimbursement caps are in place and based on three tiers of health care facilities: primary (township/community health centers), secondary and tertiary facilities. Deductibles and copayments are higher for tertiary facilities followed by secondary and primary facilities. However, because patients’ trust in township/community health centers is low, they tend to go directly to large hospitals, particularly tertiary hospitals [1].

Most inpatient and emergency services in China are delivered in public hospitals [2]. Physicians in public hospitals are salaried, and there is a rigid tenure system with tiered wage structures that are based on administrative formulas [1]. Considering health expenditure inflation under fee-for-service (FFS) reimbursement, case-based payment methods (e.g., the diagnosis-related group payment system, DRG) have recently been widely adopted in China. However, FFS reimbursement was the current method in the sample city during the period I studied.

Hospitals obtained 90% of their revenues from sales of medical services and drugs during our sample period [2]. The prices of medical services are regulated by the government; however, the price schedule charges higher than the usual cost for diagnostic tests, high-tech procedures, and drugs but lower than the usual cost for labor-intensive services (e.g., physician services).

The average reimbursement ratio for NCMS inpatient expenses has increased over time, and it reached approximately 55% in 2012 based on official statistics. However, the protection level of the NCMS is relatively low compared with that of UEBMI, which had an average reimbursement ratio of approximately 75% in 2012.

**List of abbreviations**

UEBMI: The Urban Employee Basic Medical Insurance

NCMS: New Rural Cooperative Medical Insurance Scheme

URBMI: Urban Resident Basic Medical Insurance

FFS: fee-for-service

DRG: diagnosis-related group payment system

**References:**

1. Liu, G.G., Vortherms, S.A. & Hong, X. China's Health Reform Update. *Annual review of public health*, 38(1), 431-448 (2017). <https://doi.org/10.1146/annurev-publhealth-031816-044247>
2. Barber SL, Borowitz M, Bekedam H, Ma J. The hospital of the future in China: China’s reform of public hospitals and trends from industrialized countries. *Health Policy and Planning* 29: 367–378 (2014). <https://doi.org/10.1093/heapol/czt023>
